# CENP-A nucleosomes localize to transcription factor hotspots and subtelomeric sites in human cancer cells

**DOI:** 10.1186/1756-8935-8-2

**Published:** 2015-01-13

**Authors:** Rajbir K Athwal, Marcin P Walkiewicz, Songjoon Baek, Song Fu, Minh Bui, Jordi Camps, Thomas Ried, Myong-Hee Sung, Yamini Dalal

**Affiliations:** Chromatin Structure and Epigenetics Mechanisms Unit, Center for Cancer Research, National Cancer Institute National Institutes of Health, 41 Center Drive, Bethesda, MD 20892 USA; Laboratory of Receptor Biology and Gene Expression, Center for Cancer Research, National Cancer Institute National Institutes of Health, 41 Center Drive, Bethesda, MD 20892 USA; Genetics Branch, Center for Cancer Research, National Cancer Institute National Institutes of Health, 50 South Drive, Bethesda, MD 20892 USA

## Abstract

**Background:**

The histone H3 variant CENP-A is normally tightly regulated to ensure only one centromere exists per chromosome. Native CENP-A is often found overexpressed in human cancer cells and a range of human tumors. Consequently, CENP-A misregulation is thought to contribute to genome instability in human cancers. However, the consequences of such overexpression have not been directly elucidated in human cancer cells.

**Results:**

To investigate native CENP-A overexpression, we sought to uncover CENP-A-associated defects in human cells. We confirm that CENP-A is innately overexpressed in several colorectal cancer cell lines. In such cells, we report that a subset of structurally distinct CENP-A-containing nucleosomes associate with canonical histone H3, and with the transcription-coupled chaperones ATRX and DAXX. Furthermore, such hybrid CENP-A nucleosomes localize to DNase I hypersensitive and transcription factor binding sites, including at promoters of genes across the human genome. A distinct class of CENP-A hotspots also accumulates at subtelomeric chromosomal locations, including at the 8q24/*Myc* region long-associated with genomic instability. We show this 8q24 accumulation of CENP-A can also be seen in early stage primary colorectal tumors.

**Conclusions:**

Our data demonstrate that excess CENP-A accumulates at noncentromeric locations in the human cancer genome. These findings suggest that ectopic CENP-A nucleosomes could alter the state of the chromatin fiber, potentially impacting gene regulation and chromosome fragility.

**Electronic supplementary material:**

The online version of this article (doi:10.1186/1756-8935-8-2) contains supplementary material, which is available to authorized users.

## Background

Hallmarks of the cancer state include large-scale gene expression changes [1], chromosomal rearrangement, and aneuploidy [2–6]. While the mechanistic basis for these events remains under investigation, such events have been attributed to DNA methylation changes [1], telomere disruption [7], repair and DNA damage pathway protein defects [8], replication distress [9], and misregulation of the centromere-specific histone H3 variant, CENP-A [10–13]. CENP-A’s normal function is to serve as the sole structural marker for centromeric chromatin identity [14], by directly associating with a triad of inner kinetochore proteins CENP-C, CENP-N and CENP-B [15], which in turn recruit the rest of the kinetochore and microtubules to ensure faithful genome segregation during mitosis [16]. Consequently, mislocalization of CENP-A to noncentromere regions is believed to be a prognostic marker for aneuploidies driven by chromosomal breakage and rearrangements, emanating from bicentric chromosomes [10, 11, 13, 17, 18]. Indeed, artificial overexpression studies in flies demonstrate that under certain conditions, CENP-A can seed neocentromeres [17, 19]. However, when moderately overexpressed to the levels similar to that previously seen in cancer cells [10, 11], CENP-A does not easily seed neocentromeres [20], but rather expands centromere domains [21]. In related studies, overexpressed yeast or *Drosophila* CENP-A accumulates in the euchromatic arms, where it is continually targeted for proteolysis and subsequently degraded [22, 23]. Indeed, a recent study confirms this occurs also in human HeLa cells, wherein forced artificial overexpression of tagged CENP-A results in accumulation at ectopic locations [24]. However, although CENP-A mRNA is innately overexpressed several fold in a number of human solid tumors, including colorectal tumors [10, 11, 18, 25–27], its behavior in cancer cells has not been investigated.

To elucidate consequences associated with CENP-A misregulation, we examined CENP-A mRNA and protein levels, partners, structure, and global nucleosome occupancy in human primary normal and colorectal cancers cells, as well as in primary tumors. We report that CENP-A is overexpressed at the mRNA and protein level in some human colorectal cancers. This excess CENP-A partners with histone H3, and associates with transcriptionally coupled chaperones ATRX and DAXX in colorectal cancer cell lines. This distinct class of noncentromeric CENP-A nucleosomes forms a stable octameric nucleosomal species as detected by atomic force microscopy (AFM) and confirmed by high-resolution DNA analysis, which demonstrates binding of 150 to 170 bp of DNA. These distinctive CENP-A nucleosomes localize to open regions of the genome as mapped by DNase I hypersensitivity (DHS), such as promoters of genes, and contain transcription factor binding motifs. In addition, we observe a correlation between large clusters of CENP-A and subtelomeric locations including the fragile region at 8q24. In this 8q24 region, we show that CENP-A is bound to CENP-C, a phenomena that also occurs in early human colorectal tumors, but not in normal human colon cells. Taken together, our data uncover a new role for a classical histone variant in human cancer cell lines.

## Results

### CENP-A is overexpressed, and ectopic CENP-A nucleosomes associate with H3, ATRX, and DAXX in colorectal cancer cells

**Figure 1:**
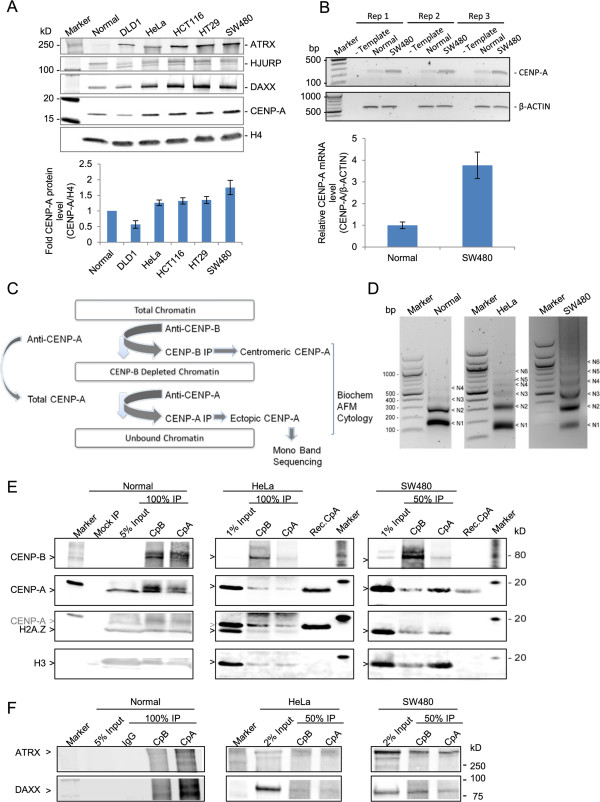
**CENP-A is overexpressed in colon cancer SW480 cells, and associates with H3, ATRX, and DAXX. (A)** Upper panel: Western blot (WB) analysis of total nuclear CENP-A, HJURP, ATRX, and DAXX, relative to core histone H4 across cell lines. Lower panel: quantification of CENP-A protein expression standardized to normal colon (replicate data from Table 1), error bars = SEM. **(B)** Upper panel: semi-quantitative PCR analysis of CENP-A mRNA expression in normal and SW480 cells in three replicates. Lower panel: quantification of the CENP-A mRNA expression normalized to β-actin from four experiments, error bars = SEM. **(C)** Scheme depicting strategy to separate centromeric from ectopic CENP-A nucleosomes and subsequent experiments performed with each fraction. **(D)** Micrococcal nuclease (MNase) ladders of input chromatin used subsequently in the primary CENP-B chromatin immunoprecipitation (ChIP) for normal, HeLa and SW480 cell lines demonstrates that mostly mono-, di-, and tri-nucleosomal arrays are present in the input chromatin. Slight variations in digestibility derive from intrinsic variability in the different cell lines’ biology. **(E)** WB slice panels show CENP-B, CENP-A, H2A.Z, and H3 protein analysis from CENP-B IP and sequential CENP-A IP from normal, HeLa, and SW480 cells. Mock immunoprecipitation (IP) indicates control immunoprecipitation with nonspecific IgG. Recombinant CENP-A (Rec. CpA) was used as a detection specificity control. Gray CENP-A arrow in third row indicates a band that was already present prior to H2A.Z probing. Data quantification is provided in Table 2. **(F)** WB for ATRX and DAXX in normal, HeLa, and SW480 CENP-B IPs versus ectopic CENP-A IPs (data summarized in Table 2).

Early reports of innate overexpression of CENP-A in colorectal tumors date back well over a decade [10]. Thus, we focused on well-characterized colorectal cancer cell lines derived from different stages of tumor progression, such as SW480, HT29, DLD-1, and HCT116, comparing them to normal colon cells. We also included HeLa cells, since they have long been used as a model for human centromere biology [28, 29]. We first examined total nuclear CENP-A protein across all the cell lines, using a sensitive fluorescence-based quantitative western blotting system (Figure 1A). Relative to normal colon cells, and standardized against internal amounts of the core histone H4, we observed CENP-A protein levels were slightly elevated in HeLa cells, lower in DLD-1, 1.35 fold overexpressed in HT29 and almost twofold overexpressed in the cell line SW480 (Figure 1A lower graph and Table 1 lists fold-values of all proteins tested in Figure 1A). To test whether the excess CENP-A protein in SW480 derived from excess mRNA, we next examined total CENP-A mRNA levels. Indeed, semi-quantitative PCR analysis indicated that CENP-A mRNA is almost fourfold overexpressed in SW480 cells compared to normal colon cells (Figure 1B*,* lower panel depicts graphical representation of 4 replicates). We next examined levels of the CENP-A chaperone Holliday junction recognition protein (HJURP), which is required for accurate loading of CENP-A to centromeres [30–32]. Surprisingly, HJURP levels do not follow those of CENP-A; the cell line possessing the most CENP-A (SW480) has normal amounts of HJURP (Table 1). This finding intrigued us because, under normal conditions, HJURP restricts CENP-A loading to centromeric nucleosomes. We wondered whether histone H3 variant chaperones were also misregulated. We assessed transcription-coupled histone chaperones ATRX and DAXX, and observed that both are overexpressed in most cancer cell lines relative to normal colon, ranging from three- to twentyfold excess protein (Figure 1A, Table 1). Thus, these data demonstrate that CENP-A gene expression is innately misregulated in some colorectal cancer cells. To examine the consequences of variable amounts of this key histone variant, we chose to focus the rest of the study on three cell lines, spanning normal (normal colon), moderate (HeLa), and high (SW480) levels of CENP-A protein.

**Table 1:** **CENP-A, ATRX and DAXX are overexpressed in colorectal cell lines**

Cell Line	CENP-A ^a^	HJURP ^a^	ATRX ^a^	DAXX ^a^
**Normal colon**	1	1	1	1
**DLD1**	0.57 ± 0.12	2.19 ± 0.73	9.40 ± 1.25	1.22 ± 0.12
**HeLa**	1.26 ± 0.09	1.55 ± 0.55	5.22 ± 0.14	2.07 ± 0.07
**HCT116**	1.32 ± 0.10	2.89 ± 0.95	13.94 ± 4.01	3.66 ± 0.15
**HT29**	1.35 ± 0.11	4.69 ± 1.80	19.05 ± 8.10	3.18 ± 0.15
**SW480**	1.75 ± 0.23	1.24 ± 0.25	22.40 ± 3.91	2.80 ± 0.22

^a^Fold-changes in each protein (CENP-A, HJURP, ATRX and DAXX) were calculated as the ratio of that protein/H4 divided by ratio of the same protein/H4 in normal colon cells. Data represents 3 replicates.

Histone variants such as H3.3/H2A.Z, which use chaperones like ATRX/DAXX, are generally excluded from centromeric CENP-A nucleosomes, and found either at pericentric regions [33] or at promoters of genes [34]. We wanted to assess potential co-occupancy of H3/H2A.Z and CENP-A, when CENP-A and ATRX are misregulated, as well as potential sites in the genome where such co-associations might occur. To enrich for potential ectopic CENP-A nucleosomes, which might be at low abundance across the genome, we first devised a scheme to enrich noncentromeric CENP-A (Figure 1C shows a brief outline of the method). We used moderately micrococcal nuclease (MNase) digested nucleosomal arrays (Figure 1D) from normal colon, HeLa, and SW480 colorectal cells. From these inputs, we sought to enrich centromere-specific CENP-A nucleosomes (henceforth referred to as ‘centromeric CENP-A’) using native immunoprecipitation (IP) for the inner kinetochore protein CENP-B. CENP-B specifically binds a motif found in most centromeric alpha satellite DNA at every alternate CENP-A nucleosome in active centromeres [35]. Gentle sequential native CENP-A IP was applied to nucleosomes left unbound (UB) from this first step in order to enrich for centromere-depleted CENP-A nucleosomes (henceforth referred to as ‘ectopic CENP-A’). While this scheme does not allow for absolute elimination of centromeric CENP-A nucleosomes (due to the fact that CENP-A can localize to autosomal alpha satellite regions lacking CENP-B box), ectopic enrichment was sufficient to examine potential differences in composition and structure using biochemical and nanomolecular tools [36]. We also performed mock IPs to ensure that background levels of histones sticking to immune-beads could be factored in for each experiment that followed. The resultant sets of IPs were then resolved on high-resolution protein gels and probed for CENP-B, CENP-A, H2A.Z, and H3 by quantitative two-color fluorescent WB. We observed a significant fraction of ectopic CENP-A present in normal colon cells, but relatively inefficient CENP-B pre-clearing (most likely due to a low abundance of extracted protein from that cell line) made the interpretation difficult (Figure 1E and Table 2). In contrast, CENP-B pre-clearing of nuclear extracts from HeLa and SW480 cancer cells was robust. Six- to seven-fold enrichment of CENP-B was observed in the CENP-B IP compared to the sequential CENP-A IP, indicating efficient centromeric chromatin depletion, leaving behind a pool of non-CENP-B associated CENP-A nucleosomes (Figure 1E and Table 2). The sequential CENP-A IP demonstrated a 3-fold enrichment of ectopic CENP-A nucleosomes compared to the centromeric fraction in SW480 cells, which constituted a 10-fold increase in comparison to HeLa, wherein ectopic CENP-A is depleted with regard to the centromeric fraction (3.02 versus 0.32 enrichment for SW480 and HeLa, respectively). Although no appreciable increase in H2A.Z was seen in the ectopic fraction of CENP-A, a threefold enrichment of canonical histone H3 in ectopic CENP-A IP was observed in SW480 compared to HeLa or normal colon cells. These data suggested co-occupancy or increased proximity of H3 and CENP-A in colorectal cancer cells (2.91 versus 0.98 H3 enrichment for SW480 and HeLa, respectively).

**Table 2:** **Protein levels of CENP-A, CENP-B, H2A.Z, H3, ATRX, and DAXX in normal and cancer cell lines**

	Normal	HeLa	SW480
**CpB fold enrichment (CpB IP/seq. CpA IP)** ^a^	1.67	5.90	7.59
**Ectopic CpA fold enrichment (Seq. CpA IP/CpB IP)** ^a^	0.54	0.32	3.02
**H2A.Z fold enrichment (Seq. CpA IP/CpB IP)** ^a^	0.87	0.87	1.08
**H3 fold enrichment (Seq. CpA IP/CpB IP)** ^a^	1.00	0.98	2.91
**ATRX fold enrichment (Seq. CpA IP/CpB IP)** ^a^	-	1.06	0.81
**DAXX fold enrichment (Seq. CpA IP/CpB IP)** ^a^	-	1.50	0.72

^a^Fluorescent western blot signals from Figure 1E and F were quantified on Odyssey Li-Cor, background-corrected, and input-adjusted fold-ratios between immunoprecipitations (IPs) were calculated as indicated.

A recent study has reported that artificially overexpressed tagged CENP-A associates with the H3.3 chaperone DAXX in HeLa cells [24]. Since both, ATRX and DAXX, were overexpressed in HeLa and SW480 cells relative to normal (Figure 1A), we next investigated association of these chaperones with centromeric and ectopic CENP-A IPs from normal colon, HeLa, and SW480 cells. We noted a strong association between these chaperones and CENP-A in colorectal cancer cells (Figure 1F, Table 2). In contrast to a recent report demonstrating that artificially overexpressed CENP-A relies on DAXX/ATRX to associate at ectopic locations, we were unable to conclude that there was specific enrichment exclusive to the ectopic CENP-A fraction, but rather noted both centromeric and ectopic CENP-A fractions associated with these transcription-coupled chaperones.

These results outline three distinguishing characteristics of the ‘high’ CENP-A state in human cells: increased association of CENP-A with H3.3 chaperones ATRX and DAXX, increased interaction of canonical H3 with ectopic CENP-A, and an abundance of the ectopic CENP-A fraction.

### Ectopic CENP-A nucleosomes have altered conformations

*In vivo*, CENP-A and H3 do not mix within single nucleosomes [37]. Given the association of ectopic CENP-A and H3 above, we were curious whether such nucleosomes, or their chromatin fibers, might present an alteration of nucleosomal features. To this end, we turned to high-resolution microscopy. In an extensive series of studies using AFM coupled to other biochemical assays, we have previously shown that in contrast to *in vitro* reconstituted recombinant CENP-A nucleosomes, which are octameric and generally indistinguishable from H3 nucleosomes [38–41], CENP-A nucleosomes purified from native human centromeres from HeLa or HEK cells, display smaller dimensions [42, 43], and attain a stable octameric height only at specific points of the human cell cycle [44]. Therefore, we next used AFM to measure native nucleosomal dimensions of ectopic versus centromeric and recombinant CENP-A nucleosomes.

**Figure 2:**
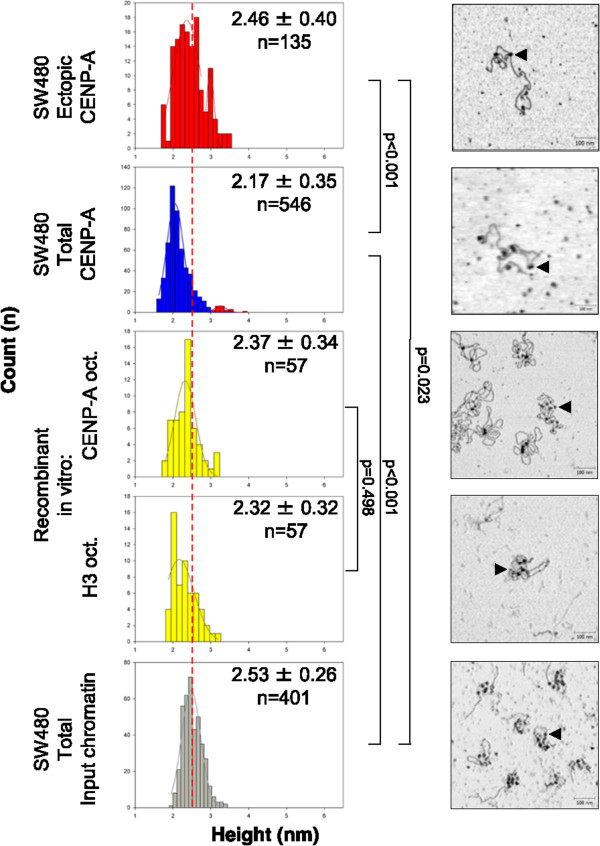
**Ectopic CENP-A forms a structurally distinct type of nucleosome in colorectal cancer cells.** Atomic force microscopy (AFM) analysis of nucleosomal heights from SW480 total input chromatin, *in vitro* reconstituted chromatin containing either H3 or CENP-A octameric nucleosomes, SW480 total CENP-A IP, and SW480 ectopic CENP-A IP. Gray graph indicates bulk octameric input, yellow indicates recombinant nucleosomes, and blue and red indicate tetrameric and octameric immunoprecipitation (IP) nucleosomes, respectively. Dashed red line indicates average octameric height. Insets contain mean ± standard deviation, and nucleosome count in each sample. *P* values are provided to the right of each dataset compared. Images show representative chromatin arrays, with arrowheads indicating single nucleosomes (scale bar 100 nm, data summarized in Table 3).

In agreement with previously published work, native bulk nucleosomes observed on extracted chromatin arrays are exclusively octameric, averaging 2.5 nm in height (Figure 2, lowest panel*,* gray, AFM data summarized in Table 3). Furthermore, *in vitro* reconstituted H3- (Figure 2*,* second panel from bottom*,* yellow*),* or CENP-A- nucleosomes (Figure 2*,* third panel from bottom*,* yellow), which are octameric [38–41, 45, 46], both possess dimensions essentially identical to bulk nucleosomes (dotted red line denotes mean octameric values). In contrast, the majority of total CENP-A nucleosomes in SW480 possess diminutive dimensions, averaging 2.1 nm in height (Figure 2, second panel from top, blue). However, upon closer examination, we noted that total CENP-A nucleosomes from SW480 have a distinct second population, with sizes reminiscent of the larger fraction of the stable octameric state (Figure 2, second panel from top, right-hand tail highlighted in red). Indeed, upon depleting centromeric CENP-A nucleosomes using the CENP-B depletion strategy above (Figure 1C), ectopic CENP-A nucleosomal arrays derived from SW480 cells display a broad height distribution with an overall average slightly smaller than bulk octamers (2.46 nm, Figure 2, top panel, red; Table 3). This broader height distribution is most likely due to partial contamination of the ectopic fraction with the centromeric CENP-A nucleosomes originating from alpha satellite arrays lacking CENP-B boxes, as mentioned above.

**Table 3:** **Ectopic CENP-A nucleosomes are stable octamers**

	SW480 bulk chromatin (n)	Recomb. H3 octamer (n)	Recomb. CENP-A octamer (n)	SW480 total CENP-A (n)	SW480 ectopic CENP-A (n)
**Height** ^a^ **(nm)**	2.53 ± 0.26 (401)	2.32 ± 0.32 (57)	2.37 ± 0.34 (57)	2.17 ± 0.35 (546)	2.46 ± 0.40 (135)
**Diameter** ^b^ **(nm)**	13.8 ± 1.4 (401)	12.1 ± 1.4 (20)	10.6 ± 1.4 (20)	14.1 ± 2.9 (546)	14.5 ± 3.0 (135)

AFM measurements of ^a^height and ^b^diameter data of ectopic versus centromeric or recombinant CENP-A nucleosomes provided as average ± standard deviation, with number of particles measured indicated in parenthesis. On average, each type of experiment has three replicates.

These data indicate that two distinct populations of CENP-A nucleosomes co-exist in colorectal cancer cells: one that contains diminutive features similar to that previously reported from native centromeres, and another that closely mimics the stable H3 or CENP-A octameric nucleosome *in vitro*.

### Ectopic CENP-A hotspots localize to DNase I hotspots and transcription factor binding sites

**Figure 3:**
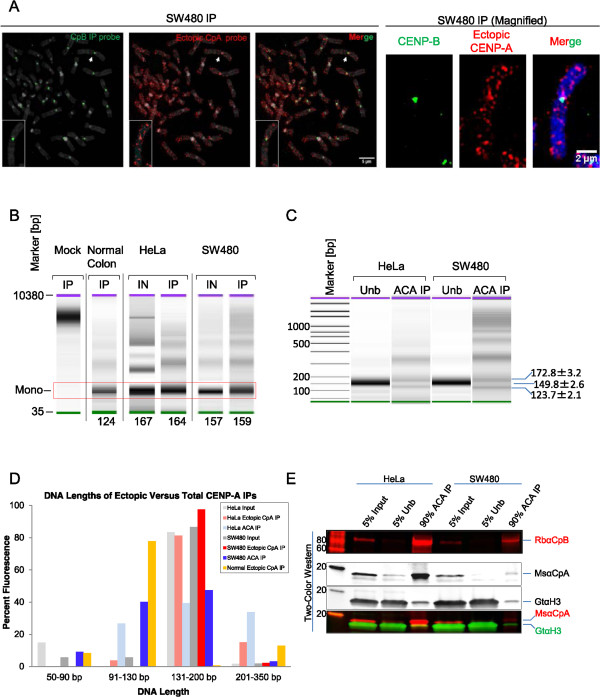
**Distribution and analysis of nucleosomal DNA associated with ectopic or total CENP-A immunoprecipitation. (A)** Fluorescent *in situ* hybridization showing location of the DNA isolated from CENP-B and ectopic CENP-A immunoprecipitations. DNA isolated from either CENP-B (green) or ectopic CENP-A IP (red) was used to generate fluorescent *in situ* hybridization (FISH) probes and hybridized to metaphase chromosomes. Chromosome indicated by white arrow is shown magnified in the right panel. Blue/grey - DAPI, scale bars: 5 μm (left panel) and 2 μm (right panel). **(B)** High-resolution Bio-Analyzer DNA chips show no detectable mononucleosomal DNA in Mock immunoprecipitation (IP) and demonstrate that ectopic CENP-A nucleosome-associated DNA is octameric in size in normal colon, HeLa, and SW480 cells, ranging from 124 to 164 bp in length. **(C)** As above, indicating the size of nucleosome-associated DNA isolated from HeLa and SW480 cells by IP with anti-centromere antibodies (ACA) serum. **(D)** High-resolution Bio-Analyzer analysis of mononucleosomal DNA values for input, ectopic CENP-A nucleosomes (from Figure 1E) and centromere-specific CENP-A nucleosomes that were purified with ACA serum (from Panel E below). DNA lengths below 350 bp were binned into four categories, and plotted by percentage of total fluorescence (pg/uL). **(E)** Two-color WB analysis of CENP-A and H3 protein levels from HeLa and SW480 isolated by IP using ACA serum.

We were curious to understand where ectopic CENP-A nucleosomes such as those above (Figures 1E, F and 2) might reside in the genome. Therefore, we amplified the nucleosomal DNA contained within SW480 CENP-B-associated centromeric and ectopic CENP-A nucleosomes, and used these two types of DNA in a co-immunofluorescence *in situ* hybridization (co-FISH) experiment against human metaphase chromosomes. As expected, CENP-B-associated nucleosomal DNA (in green) hybridizes almost exclusively to centromeres (Figure 3A). In contrast, ectopic CENP-A nucleosomal DNA (in red) hybridizes to chromosome arms (Figure 3A), illustrating the effectiveness of the CENP-B depletion strategy. This interesting distribution prompted us to generate a genome-wide map of ectopic CENP-A nucleosome residency in the genome. In order to achieve this, we performed high-throughput genome-wide sequencing using exclusively the gel-purified mono-nucleosomal fraction from thoroughly MNase digested chromatin from Mock IP DNA, or ectopic CENP-A nucleosomal DNA from normal colon, HeLa, and SW480 lines. The mono-nucleosomal fraction was first assessed using high-resolution Bio-Analyzer chips (Figure 3B and C). Whereas, no detectable DNA could be seen in the mock IP, centromeric CENP-A IPs from either HeLa cells or SW480 cells, using the classical anti-centromere antibody (ACA) (first used to identify CENP-A in human cells by the Earnshaw lab, (39)), yield two species: one at approximately 120 bp, and the other at approximately 170 bp (Figure 3C-E). The smaller species is consistent with previously published data for centromeric CENP-A nucleosomes (24, 38, 40, 43 to 50). In contrast, ectopic CENP-A mono-nucleosomes contain DNA fragments ranging from 125 to 164 bp of DNA (Figure 3B and D), greater than the 120 bp present in the CENP-A octameric crystal structure [39], or the 100-120 bp wrapping previously demonstrated to exist *in vivo* for native centromeres of yeast [47–49], *Drosophila* [42, 50] or human cells [24, 43, 44]. As suggested by the AFM data above (Figure 2), these DNA data support the possibility that ectopic CENP-A nucleosomes contain distinctive structural features.

**Figure 4:**
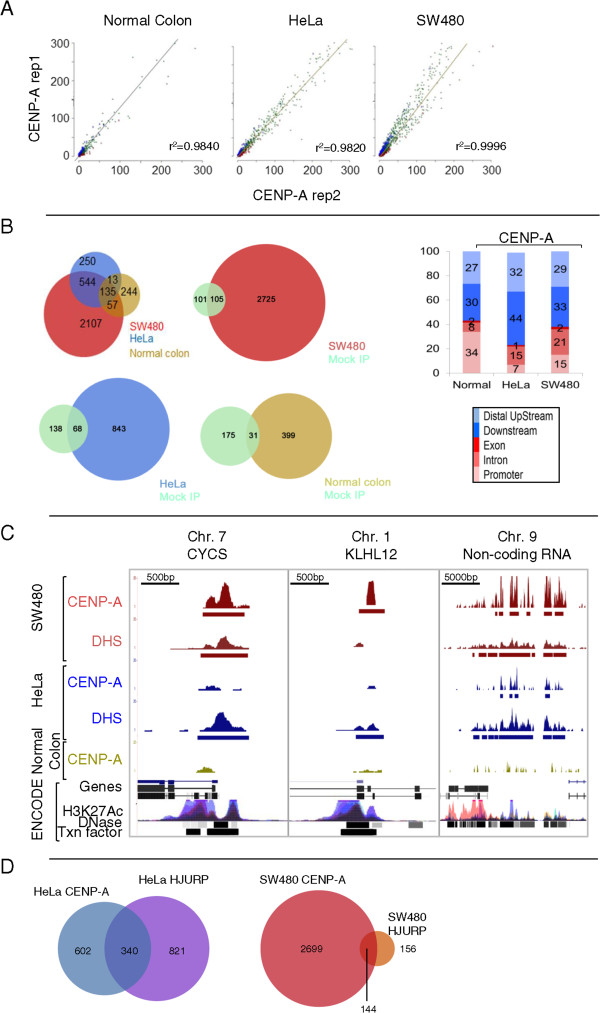
**Genome-wide analysis of ectopic CENP-A demonstrates enrichment of CENP-A hotspots at DNase I hypersensitive sites. (A)** Concordance analysis of chromatin immunoprecipitation (ChIP)-seq replicates of ectopic CENP-A IPs in normal colon, HeLa, and SW480 cells displays r^2^ > 0.9, indicative of high reproducibility. **(B)** Left*:* distribution analysis of hotspots in normal, HeLa and SW480 cells; Venn diagrams of hotspots from SW480, HeLa, or normal colon cells versus Mock immunoprecipitation (IP). Right: Genomic location of hotspots from left panel shown as a percentage of total. Legend below indicates genomic location. Fisher’s exact test was performed for comparison of HeLa and SW480 histograms: *P* = 0.0174 **(C)** Genome browser snapshots of ectopic CENP-A localization depict representative examples of CENP-A and DHS hotspots at genes overlapping with clusters of DNase I sensitivity, H3K27Acetylation and Transcription factor binding in the ENCODE data. Scale bars are in the upper left corner of each snapshot. **(D)** Distribution of HJURP hotspots in HeLa and SW480 cells. Venn diagram demonstrates 30 to 40% of HJURP hotspots overlap with ectopic CENP-A hotspots, but the number of HJURP hotspots in SW480 is very low.

Sequencing of the mononucleosomal fraction obtained from chromatin input samples from each cell line confirmed equal and robust genomic representation in the extracts, which were comparable to other ENCODE data sets (Table 4). Reassuringly, mock IP ChIP-seq performed to rule out potential background signal identified a very small number of weak background-related hotspots (approximately 200). Furthermore, correlation analyses of replicates for normal colon, HeLa, and SW480 ectopic CENP-A ChIP-seq each demonstrated excellent concordance, with an r^2^ > 0.9 for each set of replicates (Figure 4A). From the pooled replicate concordant data, we next determined statistically significant, input-adjusted tags representing true ectopic CENP-A ‘hotspots’ in the genome, at a stringent false discovery rate (FDR) of 0.1%. This method yields a robust view of CENP-A occupancy after accounting for copy number variation often found in cancer genomes. Contrary to our expectation that CENP-A would be found exclusively at centromeres or heterochromatin, ectopic CENP-A hotspots localize to noncentromeric loci in normal colon, HeLa, and SW480 cells (Figure 4B, left panel). Indeed, the main difference was the number of hotspots found in each cell line, which generally corresponded to the overall level of CENP-A expression: whereas in normal colon cells, there are approximately 450 ectopic CENP-A hotspots, in HeLa cells there is a twofold increase to approximately 950, and in SW480 cells there is an almost sixfold increase over normal colon to approximately 2,850 hotspots (Figure 4B*,* left panel). These hotspots do not arise from background signal, as only a tiny fraction of the mock IP-hotspots correlated with any of the ectopic CENP-A hotspots above (Figure 4B*,* left panel).

**Table 4:** **Genome coverage of chromatin input samples from normal colon, HeLa, and SW480**

Cell line	Number of reads	Percent genome coverage (%)
**Normal colon** ^a^	25623102	60.175
**HeLa - rep 1** ^a^	15153407	35.693
**HeLa - rep 2** ^a^	25383705	59.401
**SW480 - rep 1** ^a^	17372957	37.986
**SW480 - rep 2** ^a^	17092081	48.774

Chromatin input samples^a^ were analyzed by high-throughput sequencing and aligned to reference human genome ‘hg19’. This data was used for input-adjustment of chromatin immunoprecipitation (ChIP)-seq samples.

To investigate the nature of ectopic CENP-A hotspots, we next classified them with respect to known genomic and epigenetic features. Irrespective of the difference in the total number of hotspots, a sizeable portion of ectopic CENP-A was found at gene loci, with 23%, 38%, and 44% of ectopic hotspots at genes in HeLa, SW480, and normal colon cells, respectively (Figure 4B, right panel for histogram, and Additional file 1 contains the dataset of all hotspots discovered). Thus, CENP-A presence at genes seems to be a common feature, as it was found in all cell lines examined, with a significant fraction of those sites present at promoters of genes (7%, 15%, and 34% in HeLa, SW480, and normal colon cells, respectively). Indeed, CENP-A enrichment at promoters is statistically significant in SW480 compared to HeLa cells (Fisher’s exact test *P* value: 0.0174), suggesting that colon cells tend to accumulate CENP-A at open chromatin regions (specific examples are shown in Figure 4C).

In the experiments above, we noted that the transcription-coupled chaperones ATRX and DAXX are overexpressed in SW480 cells (Figure 1A), whereas levels of the CENP-A chaperone HJURP, which normally restricts CENP-A to centromeres [23, 31, 32, 51, 52], generally did not correlate with increased CENP-A levels. We wondered whether ectopic CENP-A accumulation at promoters is linked to HJURP presence. Therefore, we performed HJURP IPs from cross-linked chromatin, using the CENP-B depletion strategy as above (Figure 1C), followed by high throughput sequencing analysis to unveil potential sites of ectopic HJURP localization. We were unable to obtain robust ectopic HJURP enrichment. Fewer than 300 HJURP hotspots were detected in SW480 cells (Figure 4D, Additional file 1 for list of HJURP hotspots). Although 36% of the 942 HeLa CENP-A hotspots correlate with HeLa HJURP sites, only 5% of SW480 CENP-A hotspots co-localize with SW480 HJURP sites (Figure 4D). Such paucity of noncentromeric HJURP sites overlapping with ectopic CENP-A sites in SW480 is consistent with HJURP’s primary documented role as a centromere-targeted chaperone, and would support the hypothesis that overexpressed CENP-A can co-opt alternative chaperone pathways to accumulate at genes, as has recently been shown for forced overexpression of CENP-A in human cells [24].

**Figure 5:**
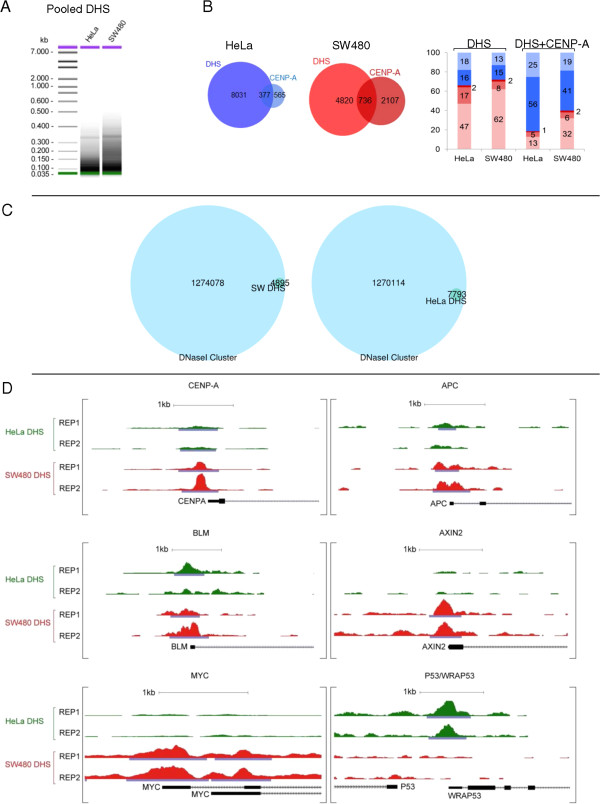
**DNase I hypersensitive sites (DHS) are enriched at promoters of oncogenes and tumor suppressor loci in the colorectal cancer genome. (A)** Sucrose gradient purification of DHS fragments resolved on High-resolution Bio-Anaylzer gels before deep sequencing. **(B)** Left: distribution analysis and overlap between HeLa DHS and HeLa CENP-A; and SW480 DHS and SW480 CENP-A. Right: Genomic locations of CENP-A hotspots coinciding with DHS shown as a percentage of total, color-coded as in **(B)**. **(C)** DHS identified in this study wholly belong to the large compendium of DHS identified by ENCODE in 129 cell lines. **(D)** Examples of DHS profiles for individual genes demonstrate that the promoters of some oncogenes are preferentially accessible in SW480 cancer cells. The gene for CENP-A itself has an increased DHS peak over its promoter in SW80 cells, correlating with increase in mRNA and protein levels for CENP-A in SW480 cells.

If CENP-A is indeed co-opting accessibility pathways to accumulate at genes, we hypothesized that chromatin accessibility might play a role in ectopic CENP-A localization. To test this, we turned to the classical DNase I nuclease hypersensitivity assay [53, 54] combined with high throughput deep sequencing [55] to pinpoint with base-pair accuracy the locations of transcription-factor bound chromatin in SW480 and HeLa cells. (Normal colon cells were present in too low density for us to reliably assess DHS sites in those cells). To release hypersensitive chromatin, we performed very light DNase I digestion of either SW480 or HeLa nuclei following established protocols [56]. DHS fragments (ranging from 50 to 350 bp) float on top of sucrose gradients, separating them from the rest of the longer DNAs, which originate from the chromatin-bound fraction. Purified DHS fragments were then subjected to deep sequencing (Figure 5A), thus generating a genome-wide distribution map of DHS.

As expected, the vast majority of DHS enrich primarily at promoters in HeLa and SW480 cells (Figure 5B*, right panel* shows histograms, and Additional file 1 for a list of DHS), and overlap completely with the compendium of aggregated DHS clusters identified by the ENCODE project for 129 human cell lines (Figure 5C). DHS identified in our data sets included promoters of housekeeping genes, oncogenes, and tumor suppressor genes (Additional file 1 for list of all DHS, examples in Figure 5D). For example, the *Myc* gene, a known regulator atop a cascade of tumor effector proteins [57], has a large DHS site astride its promoter in SW480 cells (Figure 5D). Indeed, the gene encoding CENP-A itself has a strong DHS site upstream of its promoter specifically in SW480 cells but not in HeLa cells, providing a satisfying correlation between increased accessibility of the CENP-A gene promoter, and excess CENP-A mRNA (and subsequently, protein) present in SW480 cells (Figure 5D).

When comparing DHS hotspots to ectopic CENP-A sites, we observed that a large fraction of DHS tracks with ectopic CENP-A locations (Figure 5B*,* left and middle panels). Globally, about approximately 380 CENP-A sites overlap with DHS sites in HeLa (Figure 5B, left and middle panels*),* whereas twice that number, approximately 740 SW480 CENP-A hotspots align perfectly with SW480 DHS.

A mechanistic question that arises from the correlation between ectopic CENP-A and DHS, was whether ectopic CENP-A creates DNase I sites once it binds to chromatin, or whether such sites precede CENP-A occupancy. To this end, we compared CENP-A hotspots to aggregated genome-wide locations of DHS and transcription factor binding sites from 129 and 94 cell lines respectively (ENCODE project). From these comparisons it was apparent that pre-existing DNase I and transcription factor binding sites are striking determinants of ectopic CENP-A localization (Table 5). Approximately half of normal colon or SW480 CENP-A hotspots (61% and 45%, respectively) overlap with ENCODE DNase I clusters; and a majority of normal colon and SW480 CENP-A hotspots (63% and 48%, respectively) overlap with transcription factor binding clusters found in a variety of cells (Table 5). This increase in overlap between SW480 CENP-A hotspots and our DHS analysis, compared to the ENCODE DHS data (from 26% to 45%), indicates that CENP-A can also localize to transient hypersensitive sites, which were not detected in our experiments but were captured in the vast compendium of DHS sites in the ENCODE data.

**Table 5:** **Ectopic CENP-A is enriched at DNase I hypersensitive (DHS) sites and transcription factor binding sites**

CENP-A hotspots ^a^	Normal colon	%	HeLa	%	SW480	%
**DNase clusters** ^b^	273	60.80	247	26.22	1292	45.44
**Txn Factor ChIP** ^c^	285	63.47	255	27.07	1361	47.87

Comparative analysis of ^a^CENP-A hotspots derived from normal colon, HeLa and SW480 cells compared to ENCODE data aggregates reveal a significant fraction of sites overlap with ENCODE ^b^DNase I clusters and ^c^transcription factor binding sites in the genome. Each column shows overlap in terms of number of sites or % of total CENP-A sites.

**Figure 6:**
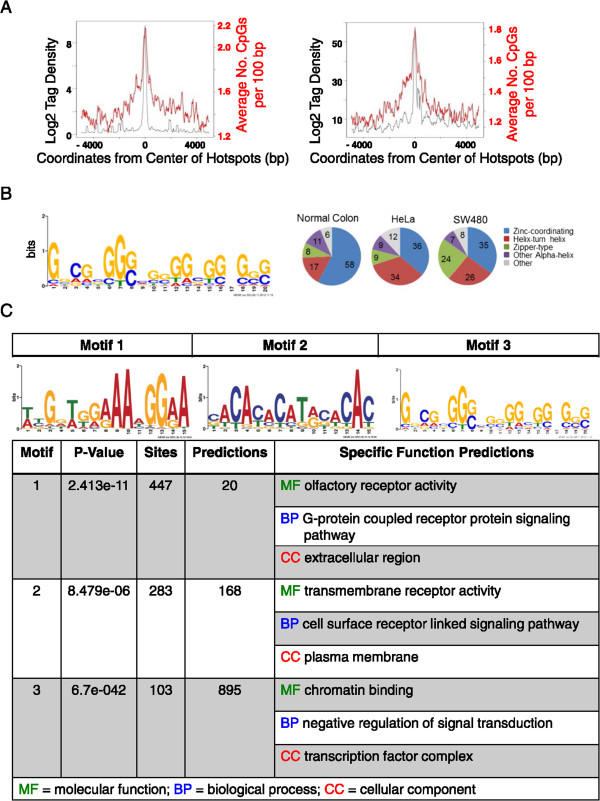
**DNA analysis of ectopic CENP-A hotspots demonstrates enrichment of CpG density and transcription factor binding motifs. (A)** Ectopic CENP-A associated DNA is moderately enriched in CpG motifs. Window shows CpG motifs enriched in a scanning 10 bp window from an overlay of all hotspots **(B)** Left: TOMTOM DNA Motif analysis of CENP-A hotspots in SW480 and HeLa cells. Right: Pie charts showing similarity of identified hotspots to known classes of transcription factor binding sites. Detailed data for analysis of hotspots can be found in Table 5. Additional file 1 contains the full list of hotspots, and a genome-wide overview is in Figure 7. **(C)** A list of other TOMTOM consensus motifs that correlate to the motifs identified in CENP-A hotspots includes chromatin effector proteins.

We were curious whether ectopic CENP-A locations had DNA sequence-specific features that might yield insights into what attracts CENP-A to them. Using the DNA consensus detection algorithm TOMTOM to detect motifs common amongst CENP-A hotspots, we discovered that CENP-A enriched sequences are not AT-rich, nor do they contain centromere-like repetitive DNA. Indeed, fewer than 20% of the hotspots contain Alu, LINE or SINE elements, and less than 0.01% of the hotspots contained centromeric consensus alpha satellite sequences (Table 6), suggesting the CENP-B depletion strategy was effective. Whether from normal colon, HeLa, or SW480, ectopic CENP-A hotspots contain CpG motifs (Figure 6A-B), and motifs associated with transcription factors, especially those of the zinc-finger and helix-turn-helix classes (Figure 6B-C). Together, the DHS and DNA motif data suggest that ectopic CENP-A accumulates at regions of high nucleosome turnover in the genome.

**Table 6:** **Analysis of total sequence tags obtained from normal colon, HeLa and SW480 CENP-A immunoprecipitations (IPs) demonstrates they are not enriched in repetitive elements**

Tags ^a^	Normal colon	%	HeLa	%	SW480	%
**Alu**	334960	0.65	226475	0.65	216285	0.63
**LINE**	10749901	20.81	6808429	19.52	6340827	18.53
**SINE**	1034138	2.00	711621	2.04	700002	2.05
**Centromeric α-satellite**	633	0.001	1163	0.003	3871	0.011
**# total tags**	51651054	100.00	34875940	100.00	34212787	100.00

Note: ^a^All sequences tags were extended to the length of 150 bp by strands to calculate overlapping.

### Ectopic CENP-A nucleosomes cluster at subtelomeric sites, including 8q24/*Myc*, in colorectal cancer cells and tumors

**Figure 7:**
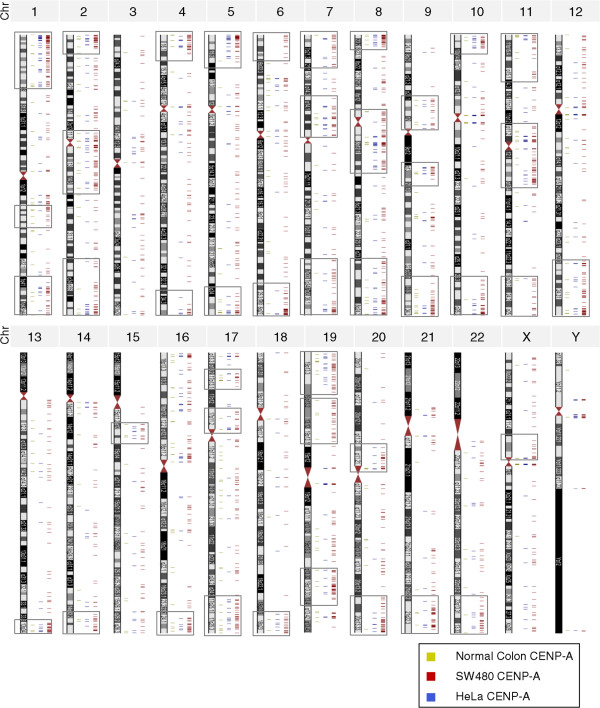
**Genome-wide distribution of ectopic CENP-A hotspots across human chromosomes.** Ectopic CENP-A clusters (gray boxes) at pericentromeric and subtelomeric regions of most human chromosomes in SW480, but not in HeLa or normal colon cells.

**Figure 8:**
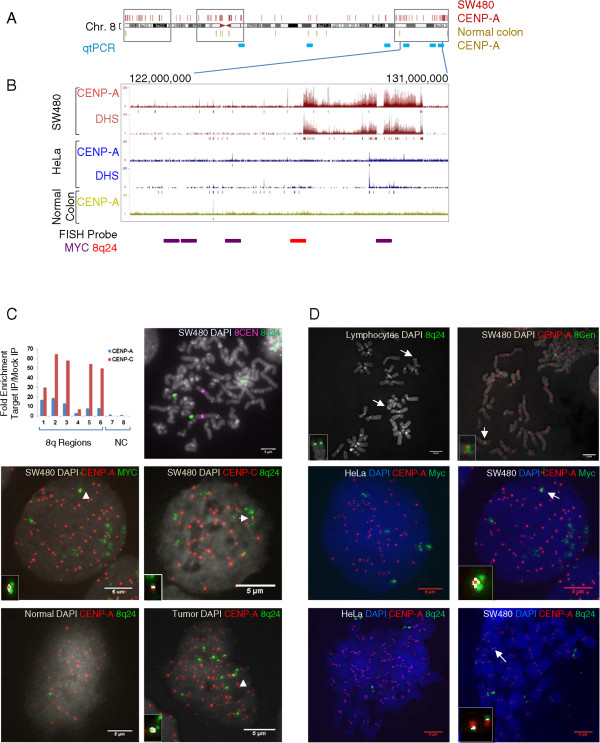
**Ectopic CENP-A clusters at a large domain at the subtelomeric 8q24/** ***Myc*** **locus in colorectal cancer cells and tumors. (A)** CENP-A hotspots cluster (indicated by gray boxes, additional examples in Figure 7) at subtelomeric regions of chromosome 8 in SW480 cells, but not in normal colon cells. **(B)** Chromatin immunoprecipitation (ChIP)-seq profiles (tag density peaks) and input-adjusted hotspot analysis (vertical bars below each profile) demonstrate CENP-A distribution relative to input chromatin upon the 30 MB domain spanning the cytogenetic band 8q24 in SW480, HeLa, and normal colon cells (note that input tag density peaks reflect increased copy number of this region in SW480 cells, but hotspot analysis takes into account copy number variation). Base numbers above indicate the genomic location, and horizontal lines below the genome browser profile indicate position of fluorescent *in situ* hybridization (FISH) probes used for FISH/co-IF in (*C*). **(C)** Upper left panel: qtPCR graph demonstrating CENP-A and CENP-C enrichment at the 8q24 locus in SW480 cells (positions of PCR primers are indicated in (A), negative control (NC) primers were selected from chromosomes 1 and 11 from regions with no CENP-A peaks based on ChIP-seq data). Upper right panel contains cytological analysis of metaphase chromosome spreads stained with FISH probe for 8q24 (in green), indicating amplification of this region in SW480 cells. One of these translocated 8q24 loci (green) associates with CENP-A (red) (middle left panel) and the inner kinetochore protein CENP-C (red) (middle right panel). Combining FISH and IF in three primary colon tumors demonstrates that CENP-A (red) co-localizes with one of the amplified regions of 8q24/*Myc* (green) in the tumor (right bottom panel) but not in normal tissue (left bottom panel). White insets and arrowheads point to co-localization spots detected by Image J automated co-localization algorithm. Quantification is provided in Table 7. **(D)** Additional controls for the FISH/IF experiments in **(C)**. First panel depicts FISH of 8q24 to metaphase spreads of normal human lymphocytes, demonstrating specific hybridization to 2 subtelomeric locations. Second panel depicts Co-IF/FISH of native centromere 8 and CENP-A on metaphase chromosome spreads in SW480 cells demonstrating that native centromere 8 still contains CENP-A and is active. Remaining panels demonstrate that CENP-A co-localizes to one of the amplified and translocated regions of 8q24/*Myc* in SW480 cells *(*right panels*)*, but not in HeLa *(*left panels*)* cells. Quantification of this data is presented in Table 7. Merge of DAPI (blue or gray), 8q24 or *Myc* (green) and CENP-A (red) is indicated for each cell line at the top of each image. Automated co-localization analysis was performed using Image J; white is indicative of co-localization shown as insets.

In the genome-wide map of all CENP-A hotspots identified in this study, we noted a qualitative clustering of CENP-A hotspots in subtelomeric and pericentromeric regions (Figure 7 shows all chromosomes, Figure 8A focuses on one example, grey boxes denote clusters). Such regions have been previously associated with chromosomal breakpoints and translocations [58]. We chose one of these domains involving the cytoband 8q24 for further analysis, as it represents one of the most frequently rearranged regions in the cancer genome of many carcinomas and hematological malignancies [59, 60]. Furthermore, this region has long been associated with tumorigenesis [61, 62], and with chromosome instability [63]. From previously published cytogenetic SKY/CGH maps, it is known that the 8q24/*Myc* locus is amplified and translocates to multiple chromosome partners in SW480 cells but not in normal colon cells [64].

The deep sequencing data uncovered a 30 MB region of CENP-A and DHS co-enrichment on the 8q24/*Myc* locus in SW480, but not HeLa or normal cells. This enrichment was apparent even after correcting for copy number amplification of this locus (Figure 8B, see input-adjusted hotspots below the tag density tracks). This result was surprising, because although this region has been extensively studied, there are no extant reports of it containing unusual histone variants. Furthermore, large domains of CENP-A usually exist only in active centromeres, wherein they attract inner kinetochore proteins such as CENP-C, which connect CENP-A to the outer kinetochore during mitosis [65]. We tested whether CENP-C was enriched in the 8q24 region. Using CENP-A and CENP-C ChIP followed by quantitative PCR (qtPCR) for probes spanning this 30 MB locus (primer locations indicated in Figure 8A), we observed robust enrichment of both CENP-A and CENP-C within the domain spanning the 8q24 locus (Figure 8C, qtPCR graph). We reasoned that a CENP-A/CENP-C domain spanning 30 MB should be visible by immunofluorescence. Therefore, we used a combination of 8q24-FISH and CENP-A-IF to visualize this region. To ensure the accuracy of detection, we first tested the 8q24 FISH probe on metaphase spreads from normal human lymphocytes. As expected, we observed two discrete subtelomeric signals per chromosome, for a total of 4 N per mitotic cell (Figure 8D*,* upper left panel). We next tested whether 8q24 was amplified and translocated in the cancer cells, as would be expected for this locus. Co-FISH for 8q24 and the native centromere 8 demonstrates one set of 8q24 signals originating from a subtelomeric location on chromosome 8, as well as a number of additional signals emanating from translocated sites (Figure 8C*,* upper right panel).

Using a combination of either 8q24 FISH and CENP-A IF, or *Myc* FISH and CENP-A IF, we next tested whether CENP-A co-localizes to any of the signals of 8q24 in normal colon, HeLa, and SW480 cells. No correlation between 8q24 and CENP-A signals can be seen in normal colon, and very little can be seen in HeLa cells (Figure 8C-D, three lower left panels). In contrast, 8q24 co-localizes to a distinct CENP-A domain in 38-66% of SW480 cells, when using either the 8q24 or *Myc* probes, respectively (Figure 8C*,* middle left panel, white arrows point to co-localized signals; Figure 8D, *lower right panels*; data are quantified in Table 7). Thus, in the colorectal cancer cell line possessing the highest amount of CENP-A protein (Figure 1A), CENP-A localizes to 8q24 in a large fraction of cells. Consistent with the qtPCR data (Figure 8C*,* first panel), CENP-C IF combined with 8q24 FISH demonstrates enrichment of CENP-C on one 8q24 locus (Figure 8C*,* middle right panel).

**Table 7:** **CENP-A is enriched at the 8q24/** ***Myc*** **locus in tumor-derived SW480 cells and in human tumors**

	Cell line/tumor	Probe	Cells with co-localization ^a^ (%)	Cells counted (N)
**Cell Line**	**HeLa**	*Myc*	5	20
**Cell Line**	**SW480**	*Myc*	66	32
**Cell Line**	**Normal Colon**	8q24	1	100
**Cell Line**	**HeLa**	8q24	8	100
**Cell Line**	**SW480**	8q24	38	100
**Tissue**	**Normal**	8q24	0	30
**Tissue**	**Tumor 1**	8q24	32.9	82
**Tissue**	**Tumor2**	8q24	36.5	85
**Tissue**	**Tumor 3**	8q24	42.9	49
**Tissue**	**Tumor 4**	8q24	78.4	88

^a^Quantification of CENP-A and 8q24/*Myc* co-localization in cancer cells and tumors after co-immunofluorescence demonstrates that a statistically significant enrichment of CENP-A occurs on one of the translocated 8q24/*Myc* loci. White spots denoting co-localization were detected using Image J’s automated co-localization algorithm.

We were intrigued by the presence of CENP-A/CENP-C at the 8q24 locus in the SW480 colorectal cancer SW480 cell line, which was derived from a late stage colorectal tumor nearly 30 years ago [64]. We sought to understand how early in tumorigenesis CENP-A might mislocalize to 8q24. We acquired primary early and late stage colorectal tumors, as well as matched normal tissue from the same patients, and performed FISH/IF to test co-localization of CENP-A to 8q24. The co-IF/FISH data show that the 8q24/*Myc* locus is amplified in all four tumors, and that CENP-A domains are enriched on one of these 8q24 loci ranging from 33 to 78% of tumor cells, depending on the donor (Figure 8C*,* lowest set of panels for representative images of normal versus tumor, white arrow points to co-localization, quantification in Table 7). Thus, CENP-A occupancy of this locus is robust and occurs even in early stage tumors.

## Discussion

In this report, we present a comprehensive examination of the histone variant CENP-A in colorectal normal and cancer cells, finding that ectopic CENP-A exists outside centromeres in human cells. Ectopic CENP-A tracks to two distinct types of domains: small regions found at promoters and accessible chromatin; and large domains found at sites of common chromosomal rearrangements. Our report yields a number of specific findings. First, CENP-A, which is innately overexpressed in cancer cells (Figure 1A-B*,* Table 1), associates with histone H3 (Figure 1E, Table 2), and shows increased association with transcription-coupled chaperones DAXX and ATRX (Figure 1F*,* Table 2). Second, ectopic CENP-A nucleosomes are stable octamers in configuration (Figure 2, Table 3), containing 125 to 165 bp of DNA (Figure 3B-D). Third, ectopic CENP-A nucleosomal tags are depleted in centromeric consensus satellite sequences (Table 6), and localize instead to unique noncentromeric locations in normal and cancer cell lines (Figure 3A). These nucleosomes occupy genes and promoters (Figure 4B-C), are HJURP-free (Figure 4D), and correlate primarily with hyper-accessible (DHS) chromatin (Figure 5, Table 5). Fourth, CENP-A/DHS ectopic sites co-occupy regions containing known transcription factor binding motifs (Figure 6, Table 5). Lastly, large clusters of CENP-A hotspots exist in regions spanning pericentric and subtelomeric regions specifically in colorectal cancer cells (Figures 7 and 8, Table 7). An example of such a cluster is at a segment of the 8q24 locus spanning the *Myc* oncogene, which, even in relatively early stage tumors tested in this study, associates with CENP-A and CENP-C (Figure 8B-D, Table 7).

A number of avenues of investigation arise from our observations. Regardless of the absolute amount of ectopic CENP-A, in normal colon cells, and in the cancer cell lines examined, there is a connection between DHS/transcription factor binding sites and ectopic CENP-A (Figures 5 and 6). That CENP-A can compete for regions linked to transcription was initially demonstrated in budding yeast, wherein CENP-A is reported to exist at barely detectable levels in a handful of genic promoters [49], which increases when CENP-A is artificially overexpressed [48]. Such CENP-A is continually targeted for subsequent proteolysis [23, 52]. Earlier work has also demonstrated that artificial constitutive overexpression of CENP-A in *Drosophila* cells results in a gradual accumulation and slow removal of CENP-A from chromosome arms [22], possibly via association with the common histone chaperone RbAp48/p55 [66]. *In vitro*, common chaperones such as p55 and NAP-1 assemble CENP-A nucleosomes efficiently [41, 66]. However, generally it has not been thought that such phenomena could occur in human cells, with many laboratories publishing studies using tagged/overexpressed CENP-A as a marker for human centromeres. However, a recent report tracking artificially overexpressed human CENP-A has demonstrated that it can occupy ectopic sites, binds histone H3.3, contains octameric size DNA fragments, and is potentially chaperoned by ATRX and DAXX [24]. Indeed, in worms, which form holocentric centromeres that line the edges of chromosomes, normal amounts of CENP-A seed centromeric domains using regions of low nucleosome turnover [67]. Our report demonstrates that a subset of native human CENP-A binds H3, forms octameric height nucleosomes, which localize to accessible chromatin domains at promoters and transcription factor sites at low levels even in normal human colon cells. This process appears to be magnified in amplitude in colorectal cancer cell lines, where a significant fraction of ectopic CENP-A nucleosomes overlap with DHS and transcription factor binding sites (Figure 5 and 6). It is feasible that a default transcription-linked pathway exists to use trace amounts of CENP-A either promiscuously expressed at the wrong time (that is, not at the end of G2 [68]), or remnant after HJURP-dependent incorporation at centromeres is complete at mid-G1 [69]. Not mutually exclusive to this explanation is the interesting possibility that defects in the timing of CENP-A expression, or promiscuous binding of CENP-A to other chaperones, coupled to defects in proteolysis, might cumulatively conspire to permit increased CENP-A accumulation at transcription factor binding sites in cells.

## Conclusions

A functional implication of stable CENP-A occupancy of promoters/DHS and its correlation with transcription factor binding sites is the potential link to gene expression changes reported in cancer cells. It is currently unknown if CENP-A is recruited by, or competes for transcription factor binding sites, either of which would be predicted to impact gene expression. Indeed, the DHS data demonstrate that many of the sites that attract CENP-A are already DHS and transcription factor binding sites, that is, high nucleosome turnover regions in a number of human cell lines. At the vast majority of genes *in vivo*, octameric H3 nucleosomes, with specific N-terminal tail modifications, dominate the epigenetic regulatory landscape [70]. Ectopic CENP-A nucleosomes would lack known H3 N-terminal tail modifications, and could potentially circumvent traditional epigenetic regulatory cascades. Thus, the functional impact of CENP-A nucleosomes on pre-existing DHS sites, or on promoter architecture, remains an exciting avenue of research. Ongoing studies are focused on whether recruitment of transcriptional activator or repressor complexes is altered in the presence of ectopic CENP-A nucleosomes, and whether such events influence gene expression patterns specifically in the cancer context.

Our study also provides support for a potential role for CENP-A in chromosomal instability. Whereas various artificial overexpression studies over the past decade have clearly established CENP-A’s ability to seed neocentromeres [17], this study provides a correlation between CENP-A and a defined chromosomal rearrangement at 8q24 in human cancer cells, which is absent in normal colon cells (Figure 8, Table 7). When normal human ES cells are challenged by induced DNA breaks, excess native CENP-A is rapidly mobilized, but does not localize to immediate break sites indicated by gamma-H2A.X staining [71]. However, a recent study used osteosarcoma-derived U2OS cancer cells, in which an artificially induced break was shown to efficiently recruit overexpressed CENP-A:GFP [72]. Thus, depending on the timing of the break, and availability of free histones, CENP-A might enrich during subsequent steps of chromatin re-establishment following repair or translocations of amplified regions in cancer cells. An avenue of research that arises from these findings is elucidating the timing of CENP-A enrichment at breakpoints during tumorigenesis, and investigating its potential role in structural rearrangements of chromosomes in subtelomeric sites such as 8q24.

Increased levels of CENP-A expression have been reported in metastatic prostate, breast, lymphoma, lung and colorectal tumors. Consequently, our observations, combined with other recently published studies on artificially induced hybrid CENP-A/H3 nucleosomes [24, 73], have implications for accumulation of downstream epigenetic defects that arise during tumorigenesis.

## Methods

### Cell culture

All cell culture medium except epithelial cell medium was supplemented with 10% fetal bovine serum and 1X penicillin and streptomycin. Cell culture media DMEM was used for HeLa cells, RPMI for SW480 and DLD1, and McCoy’s media for HCT116 and HT29. Epithelial cell medium was used to culture normal human colon epithelial cell (HcoEpiC). HcoEpiC cells are very slow growing, with the cell cycle lengths ranging from 36 to 90 hours depending on passage number.

### Total nuclear proteins extraction

Nuclei were purified from cell lines: HcoEpiC, HeLa, HCT116, DLD1, HT29 and SW480 following published procedure [43, 44]. Total nuclear proteins extracts were prepared in RIPA-Buffer. Equal amount of nuclear proteins were fractioned on SDS page gels, stained and analyzed on Odyssey and amounts were adjusted to equal amount of histone H4 for further analysis. Samples containing equal amount of histone H4 were fractioned on SDS page gel and the amounts of CENP-A, HJURP, ATRX, DAXX and histone H4 were determined by quantitative western blot analysis. Relative concentration of CENP-A in different cell lines was calculated as ratio CENP-A/H4 in a cell line divided by ratio of CENP-A/H4 in normal colon cells. Relative concentrations of ATRX, DAXX, and HJURP were calculated similarly.

### Quantitative western blot analysis

Quantitative infrared western blotting was performed using Odyssey Li-Cor CLx system (Lincoln, NE, USA). Briefly, infrared western blot (WB) signal was acquired with high dynamic range and analyzed using Image Studio software. Bands of interest were manually selected and their total intensity quantified with subtraction of median background signal from an area 3-pixels wide above and below the band in the same lane. The resulting total infrared signal values (arbitrary unit) were used for subsequent calculations as indicated.

### Chromatin immunoprecipitation

CENP-A, and CENP-B chromatin immunoprecipitation (ChIP) for WB analysis was performed following published protocol [43, 44]. Briefly, cells were harvested, washed with PBS once, PBS containing 0.1% Tween 20 three times and nuclei were released with TM2 buffer (20 mM Tris-HCl, pH 8.0; 2 mM MgCl_2_) containing 0.5% Nonidet P40 (Sigma) and 0.5 mM PMSF. The nuclei were washed with TM2 buffer to remove detergent once. To release chromatin, nuclei were digested with 0.3 U/ml MNase (Sigma) for 8 mins and reaction was stopped with the addition of 10 mM EGTA. Nuclei were extracted in low salt buffer (0.5X PBS containing 5 mM EGTA and 0.5 mM PMSF) over night at 4°C. Chromatin IP was performed using Dynabeads-protein G (Life Technologies, Grand Island, NY, USA), Agarose protein A/G plus (Santa Cruz, Santa Cruz, CA, USA) or protein G sepharose 4 Fast flow (GE Healthcare, Laurel, MD, USA) and antibodies listed below.

### Antibodies used for chromatin immunoprecipitation, western blot, and immunofluorescence

CENP-A: rabbit CENP-A (Santa Cruz; SC-22787, Santa Cruz, CA, USA); rabbit CENP-A (Millipore; 07-574, Billerica, MA, USA), custom CENP-A (CSEM laboratory); mouse CENP-A (Abcam, ab13939, Cambridge, MA, USA); goat CENP-A (Santa Cruz, sc-11277). HJURP: rabbit HJURP (Bethyl, A302-822A, Montgomery, TX, USA). H_2_A.Z: rabbit H_2_A.Z (Abcam, ab4174). CENP-C: goat CENP-C (Santa Cruz, sc11285). CENP-B: rabbit CENP-B (Santa Cruz, sc-22788), rabbit CENP-B (abcam, ac25743). ACA serum (Centromere Ab Positive Serum) BBI Solutions (Cat #SG140-2, Lot #3284-146-2, Cardiff, UK).

Chromatin IP for CHIP-Seq was performed similarly as above, except for the following modifications: MNase concentration was 0.6U/ml, digestion time was 10 min for cancer cells and 8 min for normal HcoEpiC cells, and nuclei were treated with 0.05 to 0.1% formaldehyde for gentle *in situ* crosslinking within intact nuclei for 30 min at RT, as indicated in ENCODE protocols, before extraction of chromatin in low salt buffer. IP-enriched chromatin was eluted with 100 mM NaHCO_3_ and 1% SDS at 65°C for 2 h. To reverse cross-linking, NaCl was added to the final 200 mM concentration and incubated at 67°C for additional 4 h followed by RNase A (150 μg/ml) treatment for 1 h and then proteins were digested with proteinase K (100 μg/ml) for 3 h. The DNA was purified by phenol extraction and ethanol precipitated. The DNA was repaired using PreCR Repair mix (New England Biolabs, Ipswich, MA, USA) following manufacturer’s instructions. DNA was purified using Chroma Spin columns (Clontech, Mountain View, CA, USA).

### Nucleosome reconstitution *in vitro*

Lyophilized recombinant histones (a gift from Jennifer Ottesen) were unfolded in 7 M guanidinium HCl, mixed in equimolar amounts (Either H3 or CENP-A and one each H2A, H2B, H4), and refolded into 2 M NaCl according to the protocol by Luger *et al*. [74]. Refolded nucleosomes were reconstituted onto a plasmid containing a ‘Widom 601’ positioning sequence (a gift from Carl Wu) using sequential salt dialysis adapted for low volumes. Briefly, histone octamers were mixed with plasmid DNA at 0.9:1 ratio in 2 M NaCl, 10 mM Tris-Cl pH = 8.0, 1 mM EDTA (0.18 mg/ml histones; 0.2 mg/ml DNA) and incubated on ice for 30 min. Next, 40 ul of histone/DNA mix was layered onto a dialysis disc (Millipore, 0.025um, Billerica, MA, USA) covered with a dialysis membrane (Thermo Scientific, 7000 MWCO, Waltham, MA, USA) and floated on the surface of 50 ml pre-chilled 1 M NaCl, 10 mM Tris-Cl pH = 8.0, 1 mM EDTA buffer. Sequential dialysis steps against 1 M, 0.8 M, 0.6 M, and 0.15 M NaCl (each with 10 mM Tris-Cl pH = 8.0, 1 mM EDTA) were carried out for 2 hours at 4°C (0.6 M dialysis was done overnight). Reconstituted chromatin was diluted one hundredfold in 1X PBS, 2 mM MgCl_2_ buffer and imaged on AP-mica [75].

### Atomic force microscopy imaging and analysis

AFM imaging of bulk and immunoprecipitated CENP-A chromatin was performed essentially as described previously [43, 44] with some adaptations (see manual analysis below). Extracted or IP-eluted chromatin was deposited on APS-mica (prepared as described by Dimitriadis *et al*., 2010 [43]) in the presence of divalent magnesium ions. The sample was incubated for 10 minutes, briefly rinsed with MilliQ water, and dried in a vacuum chamber. The sample was imaged using AFM 5500 (Agilent Technologies, now Keysight Technologies, Santa Clara, CA, USA) operating in AC mode (noncontact/tapping), equipped with either OTESPA or TESP silicone tip (Bruker Nano, Santa Barbara, CA, USA) with a nominal radius of 3 to 7 nm. Images were captured at 4096x4096 resolution with an instrument operating at setpoint equivalent to 65% to 75% of free amplitude (typically 1.5 to 2.5 V).

Acquired images were processed using Gwyddion (gwyddion.net) software (flattening, line correction, and polynomial background subtraction) and analyzed either manually (see below), or, for bulk controls, using NIH Image J software (imagej.nih.gov/ij/) Particle Analysis function. Briefly, the images were limited by threshold (to remove tip convolution) and filtered to include only round or elliptical shapes. Max. height, total area, and volume information was collected. SigmaPlot software was used to statistically analyze the data and generate graphs. For ectopic and recombinant CENP-A and H3 nucleosomes, manual measurements were performed in Gwyddion software to ensure that strictly DNA-associated particles were included (diameter cutoff <20 nm).

### BioAnalyzer analysis of DNA fragments obtained from chromatin immunofluorescence

DNA samples were prepared according to manufacturer’s recommendations and ran on High Sensitivity DNA Chips (Agilent Cat #5067-4626, Wilmington, DE, USA) on the Agilent 2100 BioAnalyzer system. Data with the control lower and upper limits were automatically called or manually aligned (see figure) with the Agilent 2100 Expert Software.

### DNase I digestion of chromatin from HeLa and SW480

HeLa and SW480 cells were harvested with trypsin, washed twice with ice cold PBS containing 0.1% Tween 20, and resuspend in low sucrose buffer (15 mM Tris-HCl, PH 8.0, 15 mM NaCl, 60 mM KCl, 1 mM EDTA, 0.5 mM EGTA, 1 mM spermidine, EDTA free protease inhibitors). Cells were mixed (1:1) with same buffer containing 0.04% NP-40 and nuclei were released at 4°C. Nuclei were harvested by centrifugation, washed with low sucrose buffer and DNase I digestion was performed with 20 million nuclei as previously described [76, 77]. DNA fragments of 100 to 500 bp from a chromatin digestion with 60 U/ml DNase I (Sigma, St. Louis, MO, USA) were purified using sucrose gradient [77] and DNA was precipitated in 0.1 volume of sodium acetate and 0.7 volume of isopropanol.

### Bioinformatic analysis of chromatin immunoprecipitation-seq, DNase-seq, and TOMTOM DNA motif enrichment

Purified DNA from ChIP or DNase I digested chromatin were used to prepare libraries for Illumina high-throughput sequencing as described in manufacturer’s protocol (Illumina Sequencing, San Diego, CA, USA). Libraries were sequenced to generate 35 bp single end reads using Illumina GAII sequencer at the Advanced Technology Center, NCI (Frederick, MD). Sequence reads were mapped to the reference genome hg19 by the CASAVA 1.8.2 pipeline.

### Hotspot detection for DNase-seq

We identified regions of local enrichment of sequence tags using a hotspot detection algorithm essentially as previously described [55, 77] with a false discovery rate (FDR) of 0.1%.

### Hotspot detection and input adjustment for chromatin immunoprecipitation-seq

The hotspot detection algorithm was similarly applied to ChIP-seq data with the following modification. The sequencing data from matching input samples are used for the processing of ChIP data, as a measure of background signal that might be significant. After normalizing the input data to match the number of tags in the ChIP data, the number of input tags is subtracted from the number of ChIP tags in the target window before calculating its z-score.

### DNA Motif discovery analysis

A motif discovery analysis on selected DNA sequences was performed using MEME [78] on a parallel cluster at the NIH Biowulf supercomputing facility (meme.nbcr.net/). DNA sequences for MEME input were from the top 2000 (by tag density) hotspots. To limit the computational load, only the 200-bp regions with the highest tag density were used instead of the entire width of hotspots in cases where the hotspots spanned greater than 200 bp. The width of motif for searching was set to 6 and 20 for minimum and maximum, respectively. To identify binding motifs for known transcription factors, we queried individual position-specific matrices against the Transfac database using the Tomtom software (http://meme.nbcr.net/meme/cgi-bin/tomtom.cgi). We retrieved statistically significant matches that share the majority of specific nucleotides in the sequence motifs.

### Quantitative PCR analysis

Quantitative (real time) PCR was performed using the IQ-Sybr Green Supermix kit from BioRad (#170-8880, Hercules, CA, USA) in 25 μl reaction according to the manufacturer’s protocol and samples were amplified using I-cycler fitted with MyIQ Single color real time PCR detection system (BioRad, Hercules, CA, USA). In all experiments no template- and Mock IP (normal IgG IP; negative control) controls, and input chromatin DNA and IP samples (CENP-A & CENP-C) were included from same experiment. The qtPCR reactions were setup in triplicate thus giving three threshold cycle numbers (Ct) for each sample. Experiment was repeated three separate times. Enrichments and fold changes were calculated as follows:

Ct.i = average Ct of input

Ct.m = average Ct of mock IP

Ct.IP = average Ct of IP samples (CENP-A and CENP-C)

STDV.i = standard deviation of input

STDV.m = standard deviation of mock

STDV.IP = standard deviation of IP

Step 1. Calculate ΔCt and STDV ΔCt for CENP-A, CENP-C and Mock IP using the following formulas:


Step 2. Transform ΔCt and STDV ΔCt (with respect to input) to input to linear scale fold change (FC) and fold change error (E) as follows:


Step 3 Fold Enrichment = FC of IP/ FC of Mock IP ± E

### Identification and labeling of fluorescent *in situ*hybridization probe for 8q24/*Myc*

Five overlapping bacterial artificial chromosomes (BAC) containing human chromosome 8q24 region (chr 8: 125,771,341-127,401,859) and a larger region spanning 8q24/*Myc* (location of both probes are depicted on Figure 8B) were selected and obtained from commercial source (Invitrogen, Grand Island, NY, USA). DNA was isolated from each BAC and labeled with biotin-dUTP and hybridized to normal blood lymphocytes metaphase-spread slides. Each BAC was evaluated for intensity and specificity of hybridization at target region. The BAC named RP11-150 N13 was selected to be used as a probe for 8q24 (chr 8:126,377,028 to 126,556,325), and a previously published *Myc* probe was used to confirm the results [79]. For probes, 2 μg BAC DNA was labeled with biotin-dUTP by nick translation in the presence of 4 nmol/L labeled nucleotide. Approximately 100 to 200 ng of labeled BAC probe was ethanol precipitated in the presence of 20 μg each salmon sperm DNA and human Cot1 DNA. The dry pellet was dissolved in 5 to 6 μl of hybridization buffer. The hybridization buffer contained 50% deionized formamide, 20% dextran sulfate and 4X SSC. The probe was denatured for 5 min at 80°C and then pre-annealed for 1 h at 37°C before adding to the slides for hybridization.

### Co-immunofluorescence and fluorescent in situ hybridization experiments

The IF on metaphase chromosomes, interphase cells from cell lines and tumor-normal patient sample cells, was performed on unfixed cells following published protocol with some modifications [80, 81]. Enrichment of mitotic cells was achieved by double thymidine block to arrest cells in G1 phase of cells cycle. Actively growing culture was treated 5 mM thymidine for 18 to 20 h. The cells were released from first block and grown in fresh medium for 10 h followed by second block with 5 mM thymidine for 12 h. Cells were released from second block and cultured further in fresh medium for 9 h. These cells were either harvested to make slides or treated with 100 μg/ml colcemid (Roche, Indianapolis, IN, USA) for 1 h to make metaphase chromosomes and then harvested to make slides. The cells were harvested with trypsin and washed with PBS, resuspended in 75 mM KCl, and incubated at 37°C for 13 min and then placed on ice. Cells were cytospun onto glass slides for 5 min at 600 rpm. After air drying, the slides were incubated in freshly prepared KCM buffer (120 mM KCl, 20 mM NaCl, 10 mM Tris-HCl, pH 8.0, 0.5 mM EDTA) containing 0.1% Triton X-100 and protease inhibitors (1 μg/ml aprotinin, pepstatin A, Leupeptin and antipain each) for 15 min at room temperature (RT) followed by blocking (KCM buffer containing 3% BSA, protease inhibitors and 1:100 dilution normal IgG) for 30 min and primary antibody (KCM buffer containing 1% BSA, protease inhibitors and 1:100 dilution normal IgG) for 1 h. The slides were washed with KCM three times 5 min each at RT followed by secondary antibody staining for 1 h at RT. The slides were washed with KCM buffer four times 5 min each at RT, fixed with 10% buffered formalin for 10 min at RT, washed with H_2_O three times 5 min each at RT, incubated in carnoy’s fixture for 30 min at RT followed by dehydration in ethanol series (70, 95 and 100% ethanol) 5 min each and air dried.

For FISH, slides were equilibrated in 2X SSC for 5 min and digested with pepsin (10 μg/ ml) for 3 min at 37°C. Pepsin digestion time varied for different samples based on amount of cytoplasm left after spinning cells on slides or age of slides. The slides were washed three times in 2X SSC and dehydrated in ethanol series. The DNA on slides was denatured in 70% formamide and 2X SSC at 80°C for 5 min. The slides were incubated in ice cold 70% and 95% ethanol for 3 min each followed by 100% ethanol for 5 min at RT. Then denatured slides were hybridized with pre-annealed for 20 to 24 h at 37°C. At the end of hybridization, the slides were washed in 50% formamide and 2X SSC three times for 5 min each at 45°C, 0.2X SSC four times for 5 min each at 65°C and 2X SSC at room temperature once for 5 min. After washing, slides were incubated with blocking buffer (4× SSC/0.1% Tween-20, 3% bovine albumin) containing normal sheep or goat IgG (1:100 dilution) for 1 h at 37°C. The slides were then incubated with 1:1000 dilution streptavidin alexa 488 (Invitrogen, Grand Island, NY, USA) in developing buffer (4X SSC/0.1% Tween 20, 1% BSA) containing normal IgG for 1 h. The slides were washed in 4X SSC/0.1% tween 20 solution four times at 45°C followed by two washes in 2X SSC at room temperature. Slides were air dried and mounted with aqueous mounting media containing DAPI (Vector Labs, Burlingame, CA, USA). The slides were observed with a DeltaVision RT system (Applied Precision, GE Healthcare, Issaquah, WA, USA) controlling an interline charge-coupled device camera (Coolsnap; Roper) mounted on inverted microscope (IX-70; Olympus America, Center Valley, PA, USA). Images were captured using the 100X objective at 0.06 μm z-sections, de-convolved, and 2D-projected using softWoRx (api.gehealthcare.com/api/softworx-suite.asp). One hundred interphase cells were analyzed for CENP-A and 8q24 for all cell lines. For tumors, the number of cells analyzed ranged from 70 to 85 except for one tumor in which fifty cells were analyzed due to insufficient material.

### Tumor and matched normal tissue

Tumor/normal tissues were obtained from the CHTN network. The pathology report indicated Tumor 1, Tumor 2, and Tumor 3 were moderately differentiated stage three tumor with no metastasis, high grade poorly differentiated stage three tumor with metastasis to one lymph node, and low grade well differentiated stage three tumor with no metastasis, respectively. Tumor cells were minced in buffer containing 250 mM sucrose, 15 mM Tris- HCl PH 7.5, 15 mM NaCl, 60 mM KCl, 1 mM EDTA, 0.5 mM EGTA, 0.15 mM spermine, 0.5 mM Spermidine and protease inhibitors (adapted from Dalal *et al*., 2005 [82]). Cells were collected by centrifugation at 600 g (1500 rpm) for 10 min at 4°C. The cell pellet was washed twice with same buffer. The cell pellet was resuspended in buffer containing 2 M sucrose instead of 250 mM and spun at 16,000× g for 30 min at 4°C. The cells were washed with buffer containing no sucrose and cells were cytospun onto glass slides for 5 min at 600 rpm. The slides were air dried and processed for IF and FISH as above.

## Electronic supplementary material

Additional file 1: **Genome wide chromatin immunoprecipitation (ChIP)-Seq and DNase I hypersensitivity** **(DHS)-Seq identifies CENP-A and DHS sites in colorectal cancer cells.** (A list of all hotspots identified in this study (please find attached as an xls data/spreadsheet). (XLS 6 MB)

## Abbreviations

CENP-A (-B/C/N): centromere protein A (B/C/N)
DHS: DNase I hypersensitive site
HJURP: Holliday junction recognition protein
MNase: micrococcal nuclease
ChIP: chromatin immunoprecipitation
WB: western blotting
ACA: anti-centromere antibody.
